# MSCs alleviate LPS-induced acute lung injury by inhibiting the proinflammatory function of macrophages in mouse lung organoid–macrophage model

**DOI:** 10.1007/s00018-024-05150-1

**Published:** 2024-03-11

**Authors:** Jiaqi Zhu, Jiahang Zhou, Bing Feng, Qiaoling Pan, Jinfeng Yang, Guanjing Lang, Dandan Shang, Jianya Zhou, Lanjuan Li, Jiong Yu, Hongcui Cao

**Affiliations:** 1 State Key Laboratory for the Diagnosis and Treatment of Infectious Diseases, National Clinical Research Center for Infectious Diseases, The First Affiliated Hospital, Zhejiang University School of Medicine, 79 Qingchun Rd, Hangzhou, 310003 China; 2 Laboratory Medicine Center, Department of Clinical Laboratory, Zhejiang Provincial People’s Hospital, People’s Hospital of Hangzhou Medical College, Hangzhou, 310014 China; 3 Jinan Microecological Biomedicine Shandong Laboratory, Jinan, 250117 Shandong China; 4 Department of Respiratory Disease, Thoracic Disease Center, The First Affiliated Hospital, Zhejiang University School of Medicine, 79 Qingchun Rd, Hangzhou, 310003 China; 5 Collaborative Innovation Center for Diagnosis and Treatment of Infectious Diseases, 79 Qingchun Rd, Hangzhou, 310003 China; 6 National Medical Center for Infectious Diseases, 79 Qingchun Rd, Hangzhou City, 310003 China; 7 Zhejiang Key Laboratory of Diagnosis and Treatment of Physic-Chemical Injury Diseases, 79 Qingchun Rd, Hangzhou, 310003 China

**Keywords:** Organoid–macrophage co-culture, Acute lung injury, Mesenchymal stem cell, Immunoregulation

## Abstract

Acute lung injury (ALI) is an inflammatory disease associated with alveolar injury, subsequent macrophage activation, inflammatory cell infiltration, and cytokine production. Mesenchymal stem cells (MSCs) are beneficial for application in the treatment of inflammatory diseases due to their immunomodulatory effects. However, the mechanisms of regulatory effects by MSCs on macrophages in ALI need more in-depth study. Lung tissues were collected from mice for mouse lung organoid construction. Alveolar macrophages (AMs) derived from bronchoalveolar lavage and interstitial macrophages (IMs) derived from lung tissue were co-cultured, with novel matrigel-spreading lung organoids to construct an in vitro model of lung organoids–immune cells. Mouse compact bone-derived MSCs were co-cultured with organoids–macrophages to confirm their therapeutic effect on acute lung injury. Changes in transcriptome expression profile were analyzed by RNA sequencing. Well-established lung organoids expressed various lung cell type-specific markers. Lung organoids grown on spreading matrigel had the property of functional cells growing outside the lumen. Lipopolysaccharide (LPS)-induced injury promoted macrophage chemotaxis toward lung organoids and enhanced the expression of inflammation-associated genes in inflammation-injured lung organoids–macrophages compared with controls. Treatment with MSCs inhibited the injury progress and reduced the levels of inflammatory components. Furthermore, through the nuclear factor-κB pathway, MSC treatment inhibited inflammatory and phenotypic transformation of AMs and modulated the antigen-presenting function of IMs, thereby affecting the inflammatory phenotype of lung organoids. Lung organoids grown by spreading matrigel facilitate the reception of external stimuli and the construction of in vitro models containing immune cells, which is a potential novel model for disease research. MSCs exert protective effects against lung injury by regulating different functions of AMs and IMs in the lung, indicating a potential mechanism for therapeutic intervention.

**Supplementary Information:**

The online version contains supplementary material available at 10.1007/s00018-024-05150-1.

## Introduction

Acute lung injury (ALI) is an inflammatory disease of the lungs characterized by a severe impairment of gas exchange due to an excessive inflammatory response in the lungs, disruption of the alveolar–capillary barrier, and pulmonary edema [1–3]. During lung infections, pathogens trigger the immune system, tissue-resident macrophages are activated to recruit large numbers of immune cells, and the rapid accumulation of immune cells is critical for effectively eliminating microorganisms. These recruited and activated immune cells mediate the immune response and tissue repair by producing and releasing large amounts of cytokines, chemokines, growth factors, antimicrobial substances, or adhesion molecules in the lungs [4–9]. Although the rapid response of inflammatory cells helps to fight infection, the secretion of large amounts of inflammatory factors generates a cytokine storm that dysregulates the inflammatory response, recruits excessive inflammatory cell infiltration, and creates a vicious cycle that results in severe lung injury, edema, respiratory failure, and even death [10, 11].

Clinically, the management of ALI patients is mainly mechanical ventilation with supportive therapy based on symptom improvement, which can improve the ventilation of patients, but some ALI patients continue to deteriorate with acidosis, hypercapnia, or severe refractory hypoxemia [12, 13].

Stem cell therapy is a new hope for transplantation medicine. Among stem cells, mesenchymal stem cells (MSCs) are the most commonly used cellular therapeutic agents for the treatment of immune-mediated diseases due to their immunosuppressive properties. MSCs are adult stem cells with cellular therapeutic potential due to their multidirectional differentiation potential, low immunogenicity, and immunomodulatory functions [14]. Lazarus et al. first tested MSCs as cellular drugs in human subjects in 1995 [15]. Since then, stem cell clinical trials have received extensive research attention, with hundreds of clinical trials on cellular interventions registered and published results showing that they have a safety profile along with positive efficacy [16]. MSCs have the ability to modulate inflammatory processes and have a therapeutic potential. ALI, as a typical inflammatory disease, possesses great therapeutic potential by MSCs. Current studies have shown that the mechanisms by which MSCs play their therapeutic roles are multifaceted [17, 18], and the therapeutic mechanisms of MSCs in treating ALI are worthy of further in-depth exploration and research.

In addition, in recent years, with the development of scientific advances, in vitro 3D culture technology has received widespread attention, and novel in vitro organoid models of human disease-related cells have significant applications in the fields of studying pathological mechanisms, reviewing therapeutic modalities, and drug screening [19, 20]. In 2009, researchers described an in vitro 3D organoid culture system to form simple clonal spheres of airway basal cells in vitro [21]. In recent years, lung organoids have become versatile and powerful in vitro models for studying lung diseases, but they are still rarely used in ALI-related studies. In addition, an organoid co-culture system that includes immune cells may be a good in vitro model for pathological injury processes that involve the immune system, but further studies are needed. Lipopolysaccharide (LPS), a major component of Gram-negative bacteria, can cause septicity-induced ALI and excessive inflammatory responses [22].

There are two main classes of macrophages in the mouse lungs: alveolar macrophages (AMs) and interstitial macrophages (IMs). AMs are found in the alveoli, while IMs are found between the lung epithelium and some capillaries, where they can interact with other immune cells [23, 24]. Macrophages are involved in inflammatory immune responses. In ALI mouse models, the amount of lung macrophage is elevated [3, 25]. However, the mechanism by which macrophages influence the onset and development of inflammation is still unclear. Studies to isolate the macrophage subpopulations are essential to clarify their contribution to the development of pneumonic injury. In addition, the in vitro culture of lung organoids and immune cells further promotes this goal and constitutes the most recent progress. As described subsequently in this article, we used this new approach to selectively study the functional alterations of the macrophage subpopulation during LPS-induced lung injury and to test the mechanisms by which MSCs further treat lung injury through their effects on function.

## Materials and methods

### Mouse and cell line

C57BL/6 mice with the same genetic background were provided by the Nanjing Institute of Biomedical Research of Nanjing University and housed in the specific pathogen-free grade experimental platform of the Experimental Animal Centre of Zhejiang University School of Medicine. All animal-related experimental protocols were approved by the Animal Ethics Committee of the First Affiliated Hospital of Zhejiang University School of Medicine (No. 2015-130).

293T-HA-Rspon1-Fc was used as described previously to generate a conditional medium of Rspon1 proteins [26, 27]. Stably transfected 293T cells producing mouse Rspon1–Fc fusion protein were obtained as a gift from Professor Enkui Duan and Xiaohua Lei at the Chinese Academy of Sciences.

### Isolation of primary lung organoid cells from mouse lungs

Primary lung organoid cells were isolated from adult C57BL/6 mouse lungs. After exposing the lung, blood was washed out using 1 × phosphate-buffered saline (PBS) via right ventricle injection with the inferior vena cava cutoff. Tissues were cut into small pieces and washed with pre-cooled wash solution (DMEM with 1% fetal bovine serum and 1% penicillin/streptomycin), and then transferred to pre-warmed digestion medium (DMEM with 1.5 mg/mL collagenase II and 0.5 mg/mL Dispase II) with shaking for 1.5 h at 37 °C.

During digestion, the presence of large numbers of individual cells was checked by light microscopy. Digestion was stopped using the pre-cooled wash solution and cell pellets were collected by centrifugation at 300 × g. Finally, the pellets were washed with basal medium (Advanced DMEM/F-12 with 1% GlutaMAX, 1% penicillin/streptomycin, and 10 mM HEPES) and filtered through a 70-μm filter to obtain single primary lung organoid cells.

### Culture and passage of lung organoids

The desired number of primary lung organoid cells was resuspended in matrigel after isolation, in which at least 70% of matrigel is required. The cells were placed in a 37 ℃, 5% CO_2_ incubator for 15–30 min until the matrigel solidified, and 600 μL of re-warmed mouse lung organoid complete medium (Advanced DMEM/F12 with 1% GlutaMAX, 1% penicillin/streptomycin, 10 mM HEPES, 1.25 mM N-acetylcysteine, 10% [vol/vol] Rspon1-conditioned medium, 5 mM nicotinamide, 100 ng/mL noggin, 100 ng/ml fibroblast growth factor [FGF] 10, 25 ng/ml FGF 7, and B27) was added to each well for culture. For the first 3 days after isolation, the culture medium was supplemented with a 10-μM Rho kinase (ROCK) inhibitor (Y-27632). During culture, the medium was refreshed at most every 2–3 days.

At 7–14 days after seeding, the pre-cooled basal medium was added and then pipetted up and down to disrupt the matrigel. The organoids were collected, digested into single cells by incubation with TrypLE solution (Gibco) at 37 °C, and re-seeded into new matrigel. The organoids were usually passaged with a split ratio of 1:3–5. Due to multiple passages during the cultivation of lung organoids, the passaged organoids do not contain macrophages.

### Organoid–spreading matrigel growth culture

To pre-lay the matrigel, the melted matrigel was mixed with pre-cooled basal medium in a ratio of 2:3, and a volume of 50 μL per well was encapsulated on the bottom membrane of the upper chamber of the transwell and placed in an incubator at 37 °C for 60 min to allow the matrigel to solidify. The organoids obtained from digestion by incubation with TrypLE solution (Gibco) at 37 °C were added to the upper chamber which had been coated with matrigel and the complete medium was added to grow them.

### Construction of lung organoids–immune cells model and co-culture with MSCs

AMs were derived from bronchoalveolar lavage fluid. The trachea of mice was exposed, an indwelling needle was inserted, and the lung was repeatedly irrigated with PBS solution aspirated through the hose of the indwelling needle with a 1-mL syringe, and as much fluid as possible was collected to obtain the cytosolic precipitation to isolate AMs.

IMs were derived from the lung tissue. The lung was collected and cut into pieces after heart perfusion to remove blood. The mouse Lung Dissociation Kit (Miltenyi Biotec, Bergisch Gladbach, Germany) was used for lung digestion. Filtration, density gradient centrifugation purification, and erythrocyte lysis were performed to obtain purified mouse lung immune cells. Single-cell suspensions were purified using mouse CD11b MicroBeads (Miltenyi Biotec) to collect IMs.

When the organoids on the spreading matrigel were in good growth condition, the isolated AMs or IMs were added to the upper chamber that had been coated with matrigel to achieve contact co-culture of organoids and immune cells. A complete medium solution containing 50 μg/mL LPS (Escherichia coli 0111: B4, Sigma) was added to each well of the upper chamber, and the culture was stimulated for 24 h at 37 ℃ in an incubator; the bottom of the 24-well plate was inoculated with 1 × 10^5^ MSCs in each well; the upper chamber of the lung organoids–immune cells model was placed in the 24-well plate inoculated with MSCs, and a complete medium solution containing LPS was added, and the culture was stimulated for 48 h at 37 ℃ in an incubator. The transwell upper chamber was removed, and the cells in the upper chamber were collected for subsequent analysis.

### Collection of organoids

When large and translucent organoids were observed in the matrigel, they could be applied to subsequent experiments. Pre-cooled Cell Recovery Solution (Dow Corning, Corning, NY, USA) was added, and the cell was placed on ice and shaken at 60 rpm for 1 h, so that the matrigel melted under low temperature to promote the freeing of the organoids, and attention was paid to the fact that the whole procedure should be gentle to ensure the morphological integrity of the organoids. The pipette tips and centrifuge tubes involved in this experiment should be pre-wetted with an organoid recovery anti-adhesion rinse solution to improve the recovery rate of organoids. The free organoids were washed with pre-cooled PBS. The organoids collected in this step could be analyzed subsequently.

### Immunofluorescence and immunohistochemical analyses

Lung organoids for immunofluorescence were fixed in 4% paraformaldehyde and those for immunohistochemistry were fixed in 70% ethanol.

For the immunofluorescence assay, the fixed organoids were washed in cold 0.1% Tween solution and blocked by an appropriate amount of pre-cooled PBS containing 0.1% Triton X-100 and 0.2% bovine serum albumin, transferred to labeled 1.5 mL EP tubes, and incubated on ice for 15 min. Primary antibodies were added (anti-acetyl-α-tubulin, anti-Mucin 5AC, anti-SFTPC, anti-CC10, and anti-Keratin 5) and incubated with the organoids overnight at 4 °C. After the organoids had been washed in 1 × PBS, secondary antibodies (rabbit anti-mouse IgG H&L [Alexa Fluor® 488] antibody and goat anti-rabbit IgG H&L [Alexa Fluor® 647] antibody) were added and incubated for 2 h at 4 °C. After the organoids were incubated with appropriate amount of DAPI at 60 rpm for 20 min, the organoids in the EP tubes were transferred to 24-well plates and observed by imaging under a laser confocal microscope (LSM700, Carl Zeiss, Germany).

For the immunohistochemistry assay, the fixed organoids were dehydrated and stained in 0.5% eosin (dissolved in ethanol) for at least 30 min. The organoids were washed with anhydrous ethanol, continued with xylene, and finally embedded in paraffin. Paraffin sections were stained for immunohistochemical staining, following the same processes as for normal immunohistochemical staining steps. The stained sections were scanned and analyzed using a NanoZoomer 2.0-RS scanner (Hamamatsu Photonics, Hamamatsu, Japan).

### Scanning electron microscopy and transmission electron microscopy analyses

Lung organoids for electron microscopy assay were fixed in a 2.5% glutaraldehyde solution. For the scanning electron microscopy assay, the fixed organoids were continued to be fixed with 1% osmium solution for 1 h to completely blacken the cells after washing with PBS, the cells were gradient dehydrated by adding different concentrations of ethanol solution, dried at the critical point and coated, and finally observed by scanning electron microscopy. For the transmission electron microscopy assay, the fixed organoids were successively fixed with 1% osmium solution and 2% aqueous uranyl acetate solution and gradient dehydrated by different concentrations of ethanol solution. The organoids were permeated with pure acetone, embedded using an embedding agent, left to polymerize, ultrathin sectioned, stained, and observed by transmission electron microscopy.

### Reactive oxygen species (ROS) assay

Lung organoids treated accordingly were removed from the culture chambers with cell recovery solution and washed once with culture medium. ROS levels were measured using the Reactive Oxygen Species Assay Kit (Beyotime Biotechnology, Shanghai, China). The DCFH-DA probe was diluted 1000-fold in a culture medium and added, which were incubated at 37 ℃ for 25 min, and then carefully blown every 5 min to make full contact between the probe and the cells. The lung organoids were washed three times with a culture medium to remove the residual probe. The lung organoids were observed under a confocal microscope (LSM700, Carl Zeiss, Germany).

### RNA sequencing analysis

The samples were subjected to total RNA extraction, quality check, fragmentation, reverse transcription, PCR amplification, and sequencing to obtain raw data. The raw data were subjected to subsequent sequencing quality assessment and pre-processing, genome matching and QC, protein-coding gene expression, and screening for differential genes. The number of counts of each sample gene was standardized using DESeq2 software and tested for the significance of difference, and the results were represented by the volcano and heat maps. Differentially expressed genes were characterized as those with fold-change > 2 and *P* value < 0.05. Principal component analysis (PCA) demonstrated differences between groups. Kyoto Encyclopedia of Genes and Genomes (KEGG) is the main public database on Pathways, and the KEGG database was used to perform pathway analyses of differentially protein-coding genes (combined with the results of KEGG annotations) and to calculate the significance of the differentially enriched genes for each pathway using a hypergeometric distribution test. Gene set enrichment analysis (GSEA) is a computational method for determining whether a predefined set of genes shows a statistically significant or consistent difference between two biological states. It consists of three steps, namely, calculation of enrichment scores, estimation of the significance level of enrichment scores, and correction for multiple hypothesis testing.

### Flow cytometry analysis

The lung organoid–immune cell in vitro model was digested into individual cells using Tryple solution for subsequent flow cytometry analysis. Cell viability was stained by Fixable Viability Dye eFluor780 (eBioscience, San Diego, CA, USA). The Fc receptor was blocked by an anti-mouse CD16/32 antibody (Biolegend, San Diego, CA, USA). Then cell suspensions were incubated with combinations of primary antibodies for 30 min at 4 °C. The antibodies included FITC anti-mouse CD45, APC anti-mouse iNOS, PB anti-mouse Arg-1, and PE anti-mouse MHC II. Incubation with antibodies such as Arg-1 and iNOS for the intracellular marker was performed after the Fix and Perm Kit (eBioscience). Data were acquired via CytoFLEX LX Flow Cytometer (Beckman Coulter, Inc.). Flow cytometry data analyses were conducted by FlowJo software (Tree Star, Ashland, OR, USA).

### Statistical analysis

Data were presented as means ± standard deviation (SD). Data were statistically analyzed using GraphPad Prism 8 software (GraphPad Software Inc., San Diego, CA, USA). Significance was determined by multiple *t* tests with a false discovery rate approach (*Q* = 1%) or one-way analysis of variance (ANOVA) with post hoc Tukey’s test. Flow cytometry data were analyzed using FlowJo software (Tree Star, Ashland, OR, USA). Statistical significance was considered when the *P* value was < 0.05.

### Additional methods

Western blotting (WB) procedures; real-time quantitative reverse transcription-polymerase chain reaction (qRT-PCR) procedures; and isolation, culture, and identification of MSCs of compact bone origin in mice are described in the supplementary materials.

## Results

### Construction of in vitro lung organoid model

**Fig. 1:**
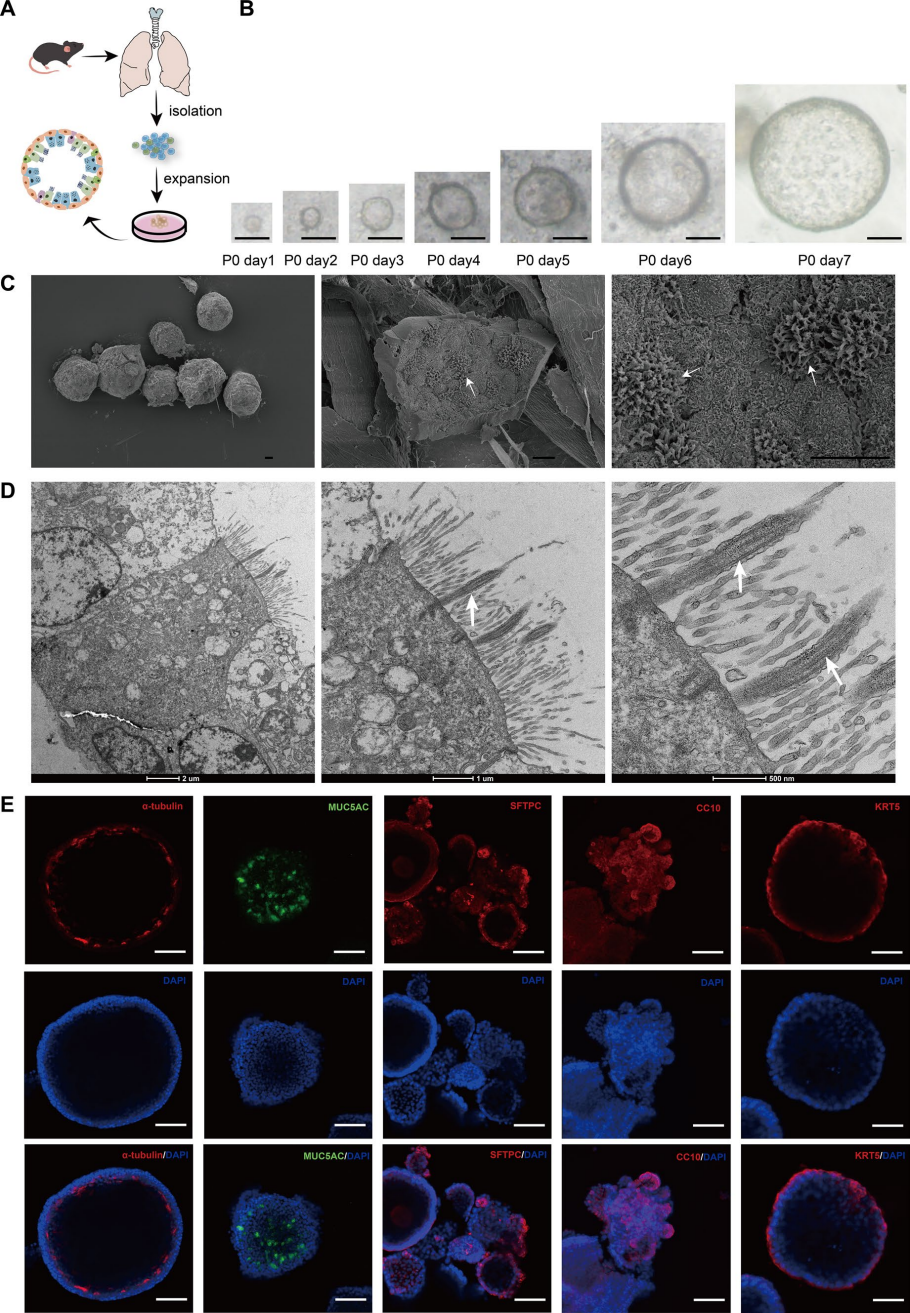
Construction and characterization of mouse lung organoids. **A** Schematic of the experimental strategy for isolation and culture of mouse lung organoids. **B** Progressive growth over time of primary (P0) lung organoids of mouse lung tissue origin (scale bar: 100 μm). **C** Representative images of mouse lung tissue-derived lung organoids taken under a scanning electron microscope. The lung organoids present intact 3D spherical structures. The white arrows indicate the ciliated structures of the lung epithelial cells observable on the ruptured inner surface (scale bar: 10 μm). **D** Representative images of lung organoids of mouse lung tissue origin taken under transmission electron microscopy, with white arrows indicating the presence of typical ciliated cells in the cultured lung organoids, and microtubule structures visible inside the ciliated cells. **E** Representative images of lung organoids of mouse lung tissue origin taken under a laser confocal microscope, where lung organoids were observed to contain multiple cell types expressing the ciliated cell-specific marker α-tubulin (red), the goblet cell-specific marker MUC5AC (green), the AT2 cell-specific marker SFTPC (red), the club cell-specific marker CC10 (red), and the basal cell-specific marker KRT5 (red) (scale bar: 50 μm)

The isolated and extracted lung cells were embedded in matrigel and under the culture of complete medium (Fig. 1A). It could be observed that rounded spherical 3D structural tissues grew in the matrigel and grew bigger and bigger in the following culture to form a huge lung organoid (Fig. 1B). The growth rate of primary organoids was slow, and after passaging, the organoids could grow rapidly and grow into larger organoids in fewer days, and the organoids could be passaged several times, and the passaged organoids still had a good growth capacity (Supplementary Fig. S1).

By scanning electron microscopy, it was observed that the cultured lung organoids had spherical 3D stereoscopic structures and the inner surface contained ciliated clusters characteristic of lung epithelial cells (Fig. 1C). Transmission electron microscopy further magnified the characteristic ciliated tissue in the lung organoids, and the typical microtubular structure in the cilia could be observed (Fig. 1D). In addition, the lung organoids were stained by immunofluorescence, and the analysis results showed that the lung organoids isolated and cultured in this study had a richer variety of cell types, including α-tubulin-expressing ciliated cells, MUC5AC-expressing goblet cells, SFTPC-expressing AT2 cells, CC10-expressing club cells, and KRT5-expressing basal cells (Fig. 1E). The results of immunohistochemistry also validated the above multiple cell types (Supplementary Fig. S2).

### Matrigel-spreading growth alters the polarization properties of lung organoids

**Fig. 2:**
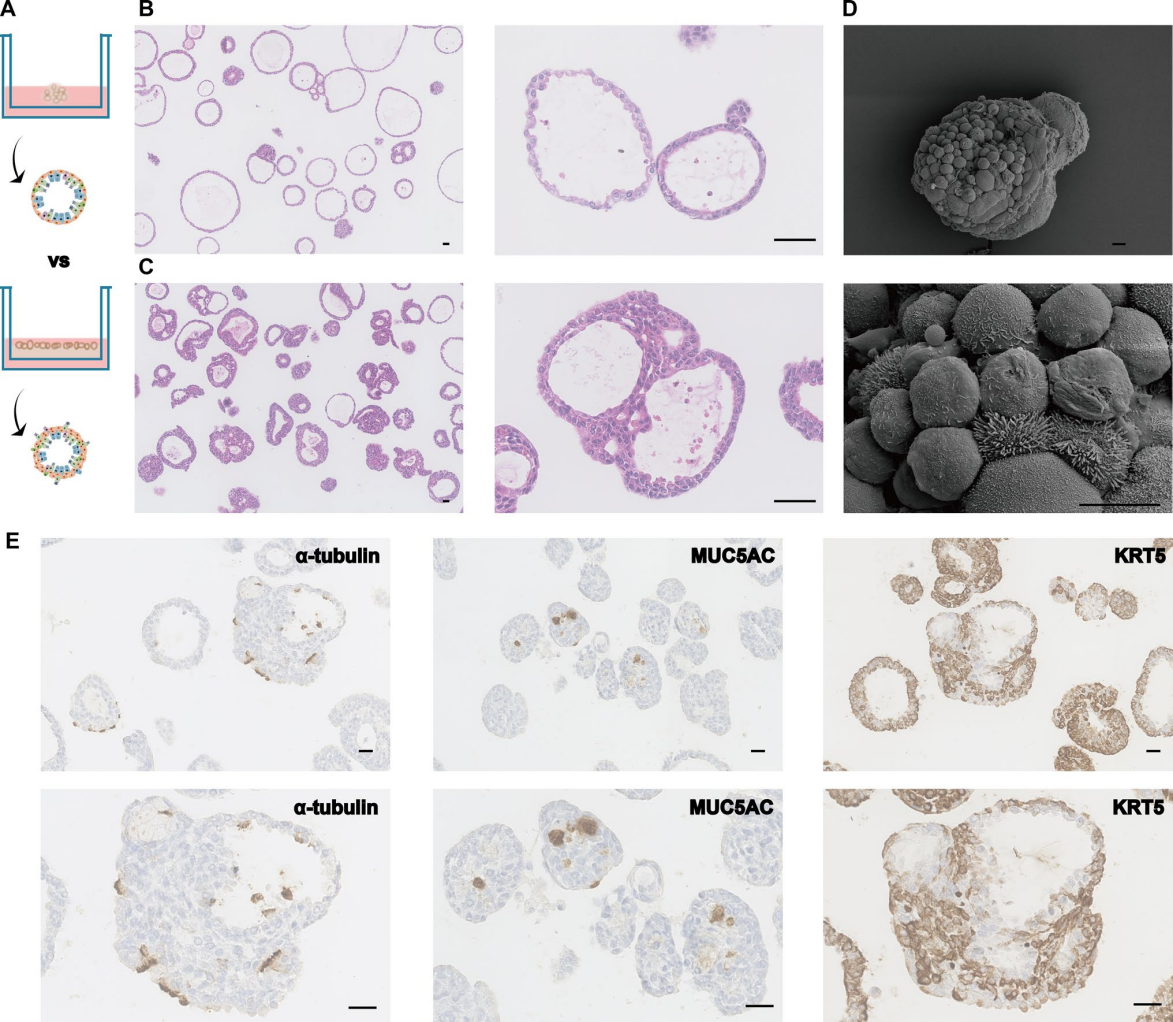
Matrigel-spreading growth alters the polarization properties of lung organoids. **A** Schematic of comparison growth pattern between mouse lung organoids wrapped in and laid on matrigel. **B** Representative image of hematoxylin and eosin (HE) staining of mouse lung organoids grown wrapped in matrigel, showing simple spherical structures (scale bar: 100 μm). **C** Representative image of HE staining of mouse lung organoids grown by matrigel spreading with thicker cell layer structure (scale bar: 100 μm). **D** Scanning electron microscope representative image of mouse lung organoids grown by matrigel spreading, with ciliated cluster structures with long cilia observed on the surface of the organoids (scale bar: 10 μm). **E** Representative image of immunohistochemical staining of mouse lung organoids grown by matrigel spreading, with ciliated cells growing outward toward the lumen and basal cells not wrapping around the periphery of the organoid alone, but showing polarized flip-flop growth features (scale bar: 50 μm)

Currently, the existing isolated lung organoids were grown embedded in matrigel, the basal layer cells were wrapped outside, and the functional cells were facing the polarized growth state of the lumen, which was not easy to contact with external stimuli, and it was often necessary to use microinjections and other methods, which were time-consuming and uncontrollable. In this study, we further improved the culture method by adopting the technique of spreading matrigel, in which the matrigel was first spread on the bottom layer, and then added into the organoids for growth and culture after solidification, and the results showed that the organoids changed their polarized growth characteristics (Fig. 2A). Hematoxylin and eosin (HE) staining results showed that the organoids grown by spreading matrigel had a thicker cell layer, which was not only the simple spherical structure when wrapped with matrigel (Fig. 2B and C). Scanning electron microscopy and immunohistochemical staining results also showed that the lung organoids grown by spreading matrigel showed polarized flip-flop growth, the basal cells shifted from the original arrangement of wrapping around the organoid surface to the lumen, and the ciliated cells that used to be oriented toward the lumen were shifted to face the outside of the lumen (Fig. 2D and E). This suggests that the lung organoids grown by spreading matrigel have better conditions to receive external stimuli, which provides a good model for subsequent studies.

### Construction of in vitro model of lung organoids co-cultured with immune cells

**Fig. 3:**
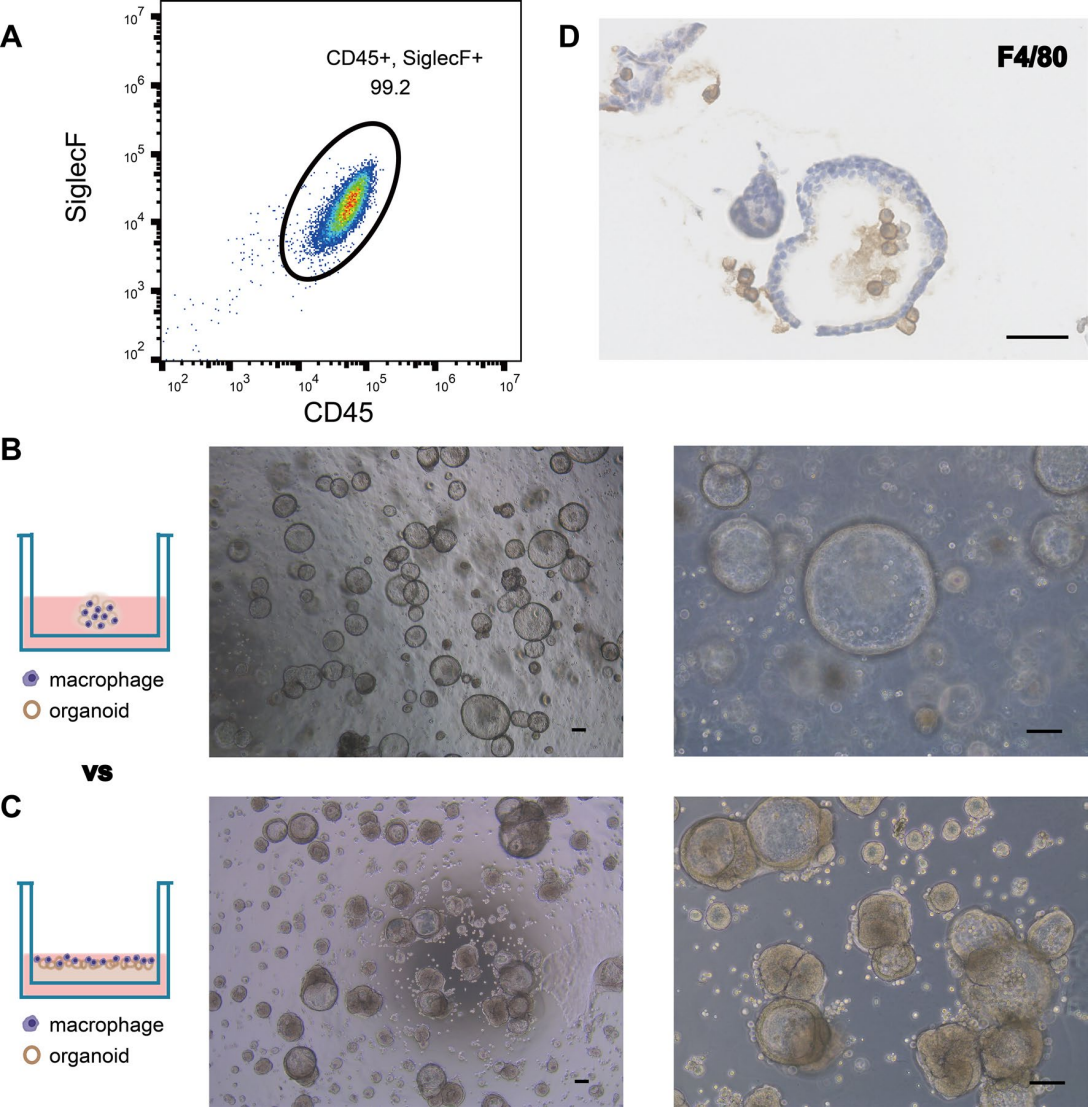
Lung organoids–immune cells model of mice grown by matrigel-spreading better simulates lung tissue structure. **A** Flow cytometry analysis to identify AMs isolated from bronchoalveolar lavage fluid. **B** Schematic diagram of lung organoids and AMs co-culture wrapped in matrigel. Representative photomicrographs of lung organoids and AMs co-culture wrapped in matrigel (scale bar: 100 μm). **C** Schematic diagram of lung organoids and AMs co-culture laid on matrigel spreading. Representative photomicrographs of lung organoids and AMs co-cultured under matrigel-spreading conditions (scale bar: 100 μm). **D** Representative image of immunohistochemical staining of lung organoids co-cultured with AMs, where F4/80-positive AMs could be present in the lumen of the organoid to better mimic the in vivo tissue structure (scale bar: 50 μm)

In lung tissue, AMs account for 90–95% of the lung’s homeostatic macrophages [28]. Due to their unique position between the lung mucosa and the external environment, AMs are essential for maintaining lung immune homeostasis and host defense, and are the first defenders of ALI, playing an important role in the initiation and progression of inflammation [29, 30]. The lung organoids constructed by co-culturing AMs with lung organoids as an in vitro model can better mimic the in vivo environment, which is important for the study of ALI. AMs are mainly found in the alveolar lumen, which can be obtained by extracting alveolar lavage fluid. The flow cytometry results showed that the AMs obtained from the isolation were relatively pure (Fig. 3A). Compared with the growth method in which the lung organoids and AMs were embedded together in the matrigel, the spreading matrigel culture reduced the blockage of the matrigel and allowed more contact between AMs and lung organoids, and round translucent AMs around the organoids could be observed (Fig. 3B and C). In addition, immunohistochemistry showed that F4/80-positive AMs could co-exist both extra- and intra-luminal of the lung organoids (Fig. 3D). Incorporation of immune cells in co-cultures so that organoids can better mimic in vivo tissue structures has been a hot research topic in recent years. The lung organoid–AM model constructed in this study provides a good model for the in vitro study of acute lung injury and the treatment of acute lung injury with MSCs.

### Culture and identification of MSCs

The isolated MSCs derived from mouse compact bone were grown adherently in culture dishes, and it was observed that the cells were spindle shaped or fusiform (Supplementary Fig. S3A). The 3rd to 5th generation cells were used to identify the induced differentiation of bone, lipid, and chondroblast (Supplementary Fig. S3B–D) and to detect the expression of specific markers (Supplementary Fig. S4). These cells would be used in subsequent experiments.

### MSCs modulate AMs to inhibit inflammatory responses in lung organoids

**Fig. 4:**
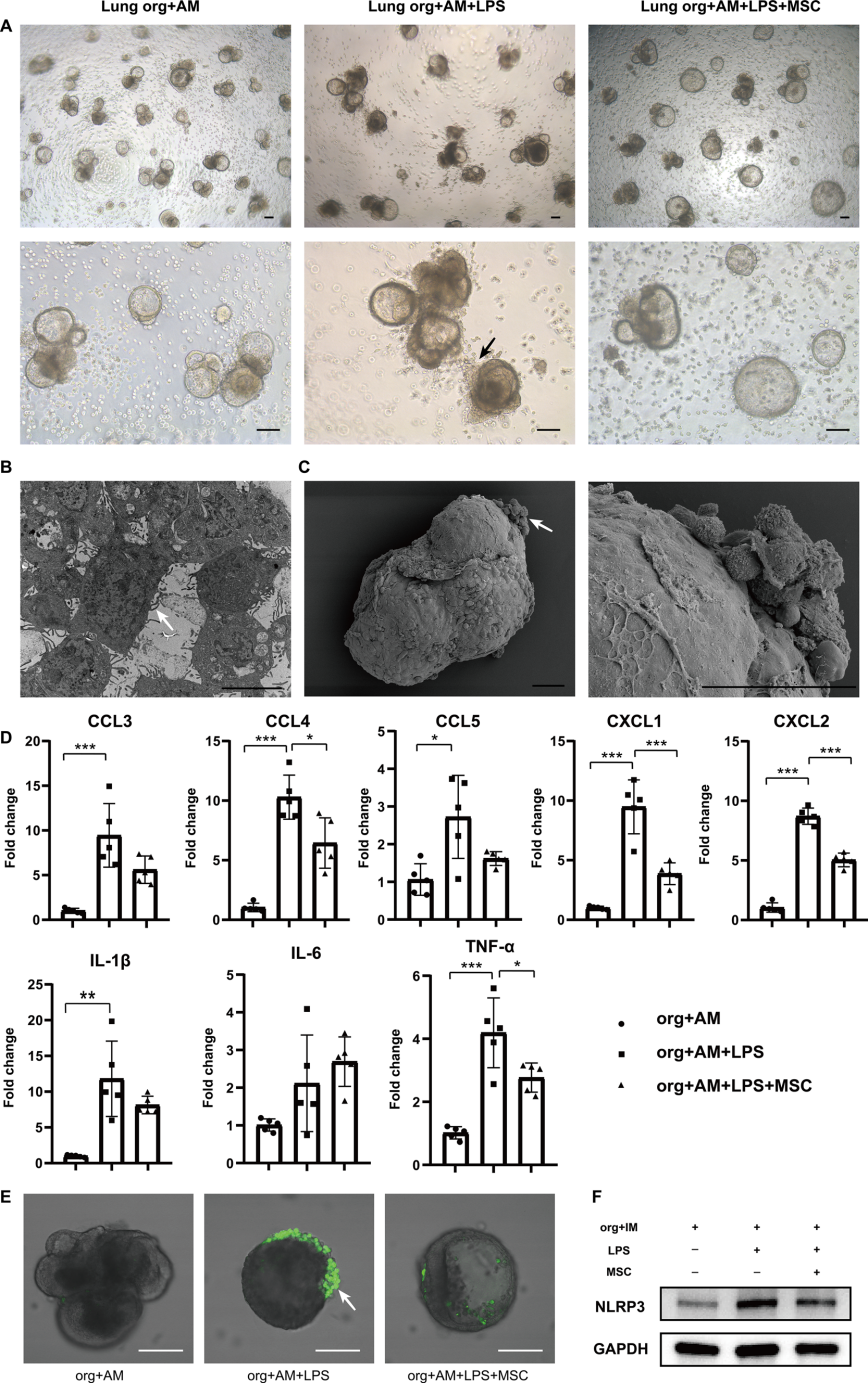
MSCs suppress lung organoids’ inflammatory responses by modulating AMs. **A** Representative photomicrographs of lung organoids–AMs, lung organoids–AMs subjected to LPS stimulation for 72 h, and lung organoids–AMs subjected to lipopolysaccharide (LPS) stimulation for 72 h while co-cultured with MSCs under the condition of matrigel spreading (scale bar: 100 μm). The black arrows indicate AMs aggregating toward lung organoids under LPS stimulation. **B** Representative image of transmission electron microscopy of lung organoids–AMs after 72 h of LPS stimulation (scale bar: 5 μm). The white arrows indicate AMs. **C** Representative scanning electron microscopy images of lung organoids–AMs after 72 h of LPS stimulation (scale bar: 50 μm). The white arrows indicate AMs. **D** Statistical plots of mRNA levels of CCL3, CCL4, CCL5, CXCL1, CXCL2, IL-1β, IL-6, and TNF-α expression in lung organoids–AMs with LPS stimulation and co-culture with MSCs (*n* = 5, *P < 0.05, **P < 0.01, ***P < 0.001). **E** Representative graphs of ROS detection in lung organoids–AMs subjected to LPS stimulation for 72 h while co-cultured with MSCs (scale bar: 50 μm). The white arrows indicate AMs showing green fluorescence, with high expression of ROS. **F** Representative images of western blotting of NLRP3 in lung organoids–AMs subjected to LPS stimulation for 72 h while co-cultured with MSCs

Further, an in vitro model of lung injury induced by LPS-stimulated lung organoids–AMs was employed. After LPS stimulation, the lumen of lung organoids was no longer translucent, the lumen became darker, and the number of organoids with cellular clusters increased significantly, suggesting that the cellular status was gradually deteriorating, whereas the cellular status was improved in the MSC co-culture group (Fig. 4A). In addition, LPS stimulated AMs to undergo obvious chemotaxis and aggregation phenomenon, and it was observed that AMs scattered in the interstitial space of the organoids were obviously reduced and gradually aggregated around the lung organoids (Fig. 4A). Transmission electron microscopy showed that the lung organoids contained a large number of AMs protruding from the surface of the cells (Fig. 4B), and scanning electron microscopy showed that obvious aggregated clusters of AMs appeared on the surface of the organoids (Fig. 4C). In contrast, MSC co-culture treatment significantly alleviated the chemotactic aggregation of AMs toward the lung organoids, and a large number of AMs still existed in the interstitial space of the organoids, and the lumen of the organoids was more translucent. The above results suggested that MSCs had an inhibitory effect on the chemotaxis of AMs in organoid injury induced by LPS stimulation.

Dysregulation of inflammatory response is one of the important factors causing ALI. We further explored that there was a significant increase in inflammation levels of lung organoids–AMs after LPS stimulation, and the levels of CCL3, CCL4, CCL5, CXCL1, CXCL2, interleukin-1β (IL-1β), and tumor necrosis factor-α (TNF-α) were significantly upregulated, while MSCs suppressed the expression of most of the inflammatory chemokines (Fig. 4D). Interestingly, MSCs significantly inhibited ROS production in AMs (Fig. 4E). High levels of ROS could lead to the activation of NOD-like receptor thermal protein domain-associated protein 3 (NLRP3) inflammasome, a key proinflammatory factor that triggers inflammatory responses. Further examination of the expression level of NLPR3 in lung organoids showed that MSCs could significantly inhibit the expression of NLRP3 (Fig. 4F). Therefore, using an in vitro model of lung organoids–AMs, we revealed that MSCs play a key role in suppressing inflammatory responses, inhibiting AM chemotaxis, reducing ROS production, and lowering NLRP3 inflammasome levels.

### MSCs modulate IMs to inhibit inflammatory responses in lung organoids

**Fig. 5:**
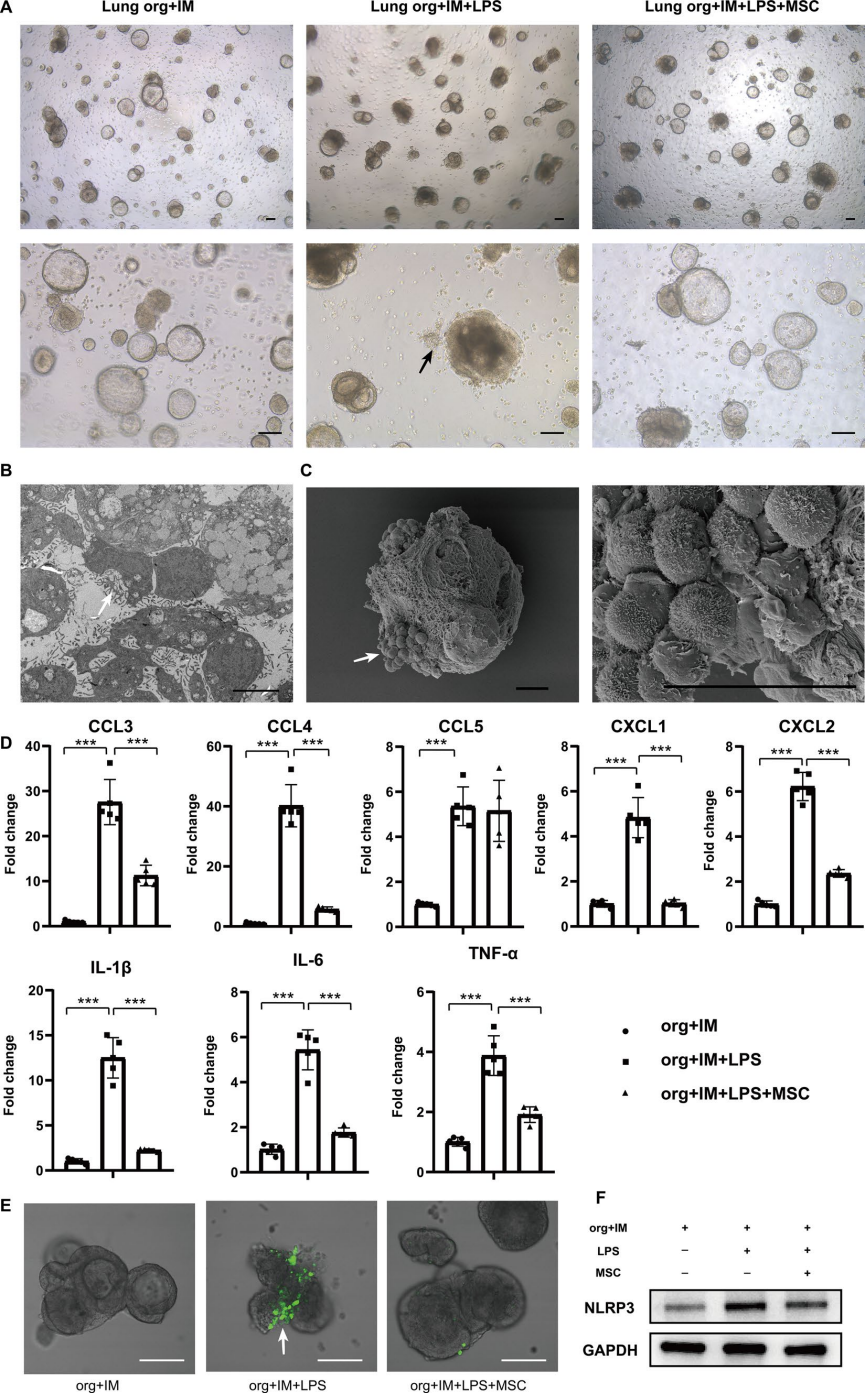
MSCs suppress lung organoids’ inflammatory responses by modulating IMs. **A** Representative photomicrographs of lung organoids–IMs, lung organoids–IMs subjected to LPS stimulation for 72 h, and lung organoids–IMs subjected to LPS stimulation for 72 h while co-cultured with MSCs under the condition of matrigel spreading (scale bar: 100 μm). The black arrows indicate IMs aggregating toward lung organoids under LPS stimulation. **B** Representative image of transmission electron microscopy of lung organoids–IMs after 72 h of LPS stimulation (scale bar: 5 μm). The white arrows indicate IMs. **C** Representative scanning electron microscopy images of lung organoids–IMs after 72 h of LPS stimulation (scale bar: 25 μm). The white arrows indicate IMs. **D** Statistical plots of mRNA levels of CCL3, CCL4, CCL5, CXCL1, CXCL2, IL-1β, IL-6, and TNF-α expression in lung organoids–IMs with LPS stimulation and co-culture with MSCs (*n* = 5, *P < 0.05, **P < 0.01, ***P < 0.001). **E** Representative graphs of ROS detection in lung organoids–IMs subjected to LPS stimulation for 72 h while co-cultured with MSCs (scale bar: 50 μm). The white arrows indicate IMs showing green fluorescence, with high expression of ROS. **F** Representative images of western blotting of NLRP3 in lung organoids–IMs subjected to LPS stimulation for 72 h while co-cultured with MSCs

During homeostasis, there are two main types of macrophages in the mouse lung: In addition to the aforementioned AMs residing in the alveolar space, there is also a group of CD11b^hi^ macrophage occupying the lung interstitium. CD11b^hi^ macrophages, often referred to as IMs, are limited to the lung interstitium. Their close association with other cells in the interstitium and the extracellular matrix implies that they may possess a more significant regulatory role and act as a secondary line of defense for the lung [31, 32]. We further isolated CD11b-positive cells from mouse lung tissues and co-cultured them with lung organoids to explore their functional changes during MSC treatment of ALI. The results showed that IMs exhibited similar properties to AMs, and LPS stimulation also led to IMs converging and aggregating around the lung organoids, which were also in a worse state, with the lumen no longer translucent and darker and with aggregated cell clusters, which were alleviated by MSCs (Fig. 5A–C). Inflammation levels of LPS-stimulated lung organoids–IMs also showed a significant increase, while MSCs suppressed the upregulation of CCL3, CCL4, CXCL1, CXCL2, IL-1β, IL-6, and TNF-α expression (Fig. 5D). Similarly, MSCs significantly inhibited ROS production in IMs (Fig. 5E) and significantly suppressed the expression of NLRP3 (Fig. 5F). The above results suggest that MSCs can inhibit the chemotaxis and inflammatory responses of IMs in response to LPS-stimulated organoid injury.

### MSCs inhibit LPS stimulation-induced inflammatory responses in AMs through the NF-κB pathway

**Fig. 6:**
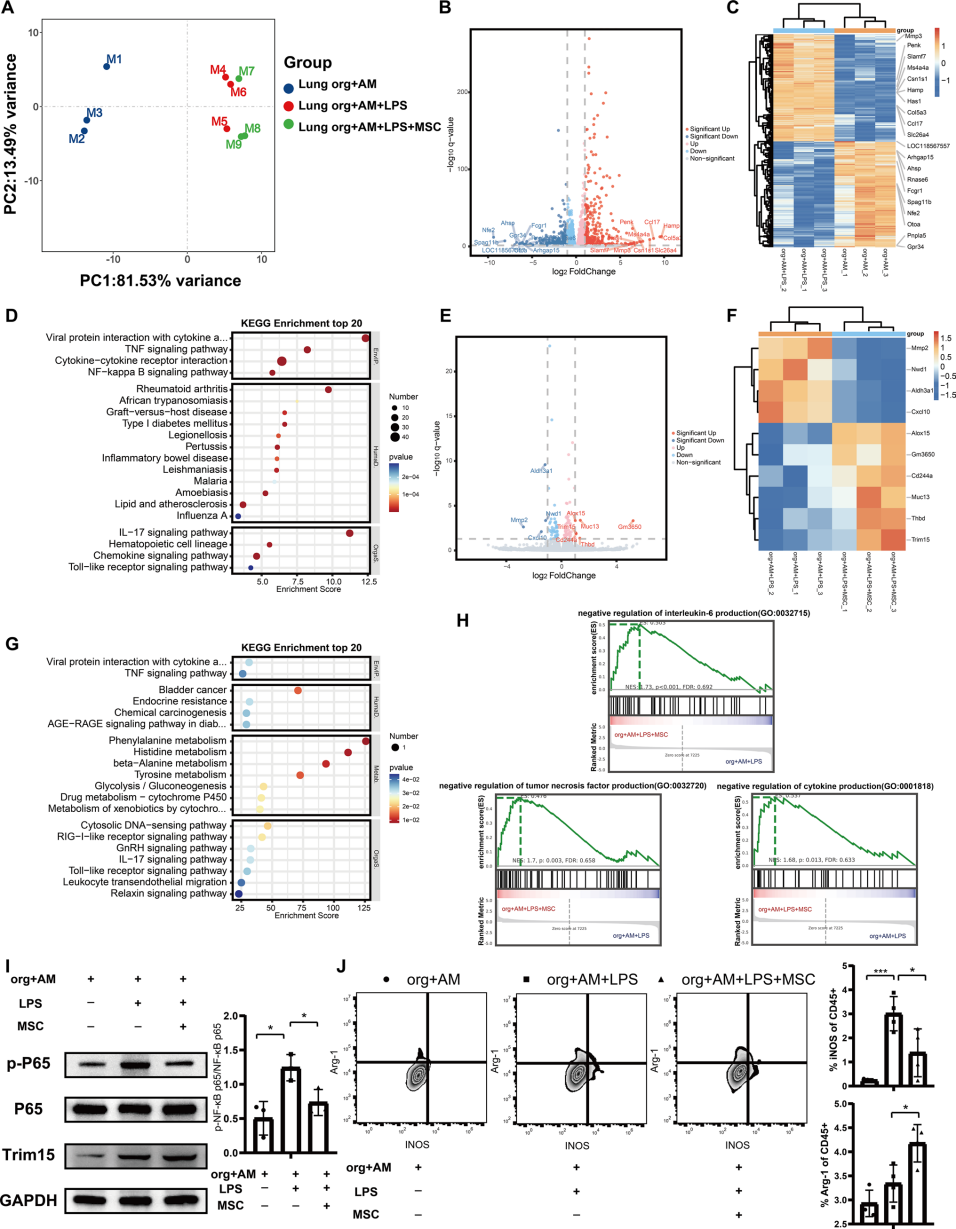
MSCs inhibit LPS stimulation-induced inflammatory responses in AMs through the NF-κB pathway. **A** PCA plots of transcriptome expression in the lung organoid–AM group, LPS lung organoid–AM group, and LPS/MSC lung organoid–AM group. **B** Volcano plot for DEGs between lung organoid–AM group and LPS lung organoid–AM group. **C** Heatmap for DEGs between lung organoid–AM group and LPS lung organoid–AM group. **D** KEGG pathway enrichment analysis of the upregulated genes of lung organoid–AM group after LPS stimulation. **E** Volcano plot for DEGs between LPS lung organoid–AM group and LPS/MSC lung organoid–AM group. **F** Heatmap for DEGs between LPS lung organoid–AM group and LPS/MSC lung organoid–AM group. **G** KEGG pathway enrichment analysis of the downregulated genes of lung organoid–AM group after LPS stimulation by MSC treatment. **H** GSEA of DEGs of the downregulated genes of lung organoid–AM group after LPS stimulation by MSC treatment. **I** Representative images of western blotting of NF-kB P65, p-NF-kB P65, and TRIM15 in lung organoids-AMs subjected to LPS stimulation for 72 h while co-cultured with MSCs. Quantitative analysis of the relative expression level of p-NF-κB P65 and NF-κB P65 (*n* = 3). **J** Expression of intracellular iNOS (M1 macrophage marker) and Arg-1 (M2 macrophage marker) in AMs after being treated with various formulations analyzed by flow cytometry (*n* = 4). Data are expressed as mean ± SD. *P < 0.05, ***P < 0.001

To further understand the therapeutic effects of MSCs at the molecular level, we quantified transcriptional changes in the in vitro lung organoids–immune cells co-culture model using RNA-seq technology. The results using PCA showed significant differences in transcriptome expression patterns among the three groups: lung organoid–AM group, LPS lung organoid–AM group, and LPS/MSC lung organoid–AM group (Fig. 6A). Compared with the lung organoid–AM group without LPS stimulation, the LPS-stimulated group had 384 upregulated differentially expressed genes (DEGs) and 380 downregulated DEGs, as shown in the volcano and heatmaps (Fig. 6B and C). Genes associated with bacterial response, immune response, or inflammatory response such as Penk, Slamf7, and Ccl17 were upregulated, and Rnase6 encoding an antimicrobial peptide was downregulated. KEGG enrichment analysis showed that the upregulated genes were enriched in many pathways associated with the immune system or inflammatory pathways, including the TNF signaling pathway, NF-kappa B signaling pathway, IL-17 signaling pathway, and Toll-like receptor signaling pathway (Fig. 6D). Six upregulated genes and four downregulated genes emerged after co-culture treatment with MSCs (Fig. 6E and F). Further investigation of the potential pathways for downregulation of the differentially expressed genes revealed that KEGG was also enriched in many immune and inflammatory pathways, including TNF signaling pathway, IL-17 signaling pathway, and Toll-like receptor signaling pathway (Fig. 6G). GSEA results showed that after co-culture of MSCs, genes were enriched in negative regulation of interleukin-6 production, negative regulation of tumor necrosis factor production, and negative regulation of cytokine production (Fig. 6H). In addition, it has been shown in the literature that TRIM15 among the upregulated genes is a TNF-α-induced late response gene that inhibits the TNF-α-induced NF-κB pathway and is a feedback regulator that controls the proinflammatory NF-κB pathway [33]. The WB results confirmed that MSCs inhibited the LPS-induced decrease in TRIM15 expression and reduced NF-κB phosphorylation (Fig. 6I). NF-κB is a major transcriptional factor driving M1 polarization, and its activation and nuclear translocation drive transcription of genes encoding cytokines and chemokines [34]. Flow cytometry analysis showed a significant increase in the proportion of M1 in AMs after LPS stimulation, whereas MSCs inhibited the increase in the M1 percentage and promoted the increase in the M2 percentage (Fig. 6J). These results suggest that MSCs inhibit the inflammatory response induced by LPS stimulation in AMs through the NF-κB pathway.

### MSCs inhibit LPS stimulation-induced inflammatory responses and MHC II in IMs via the NF-κB pathway

**Fig. 7:**
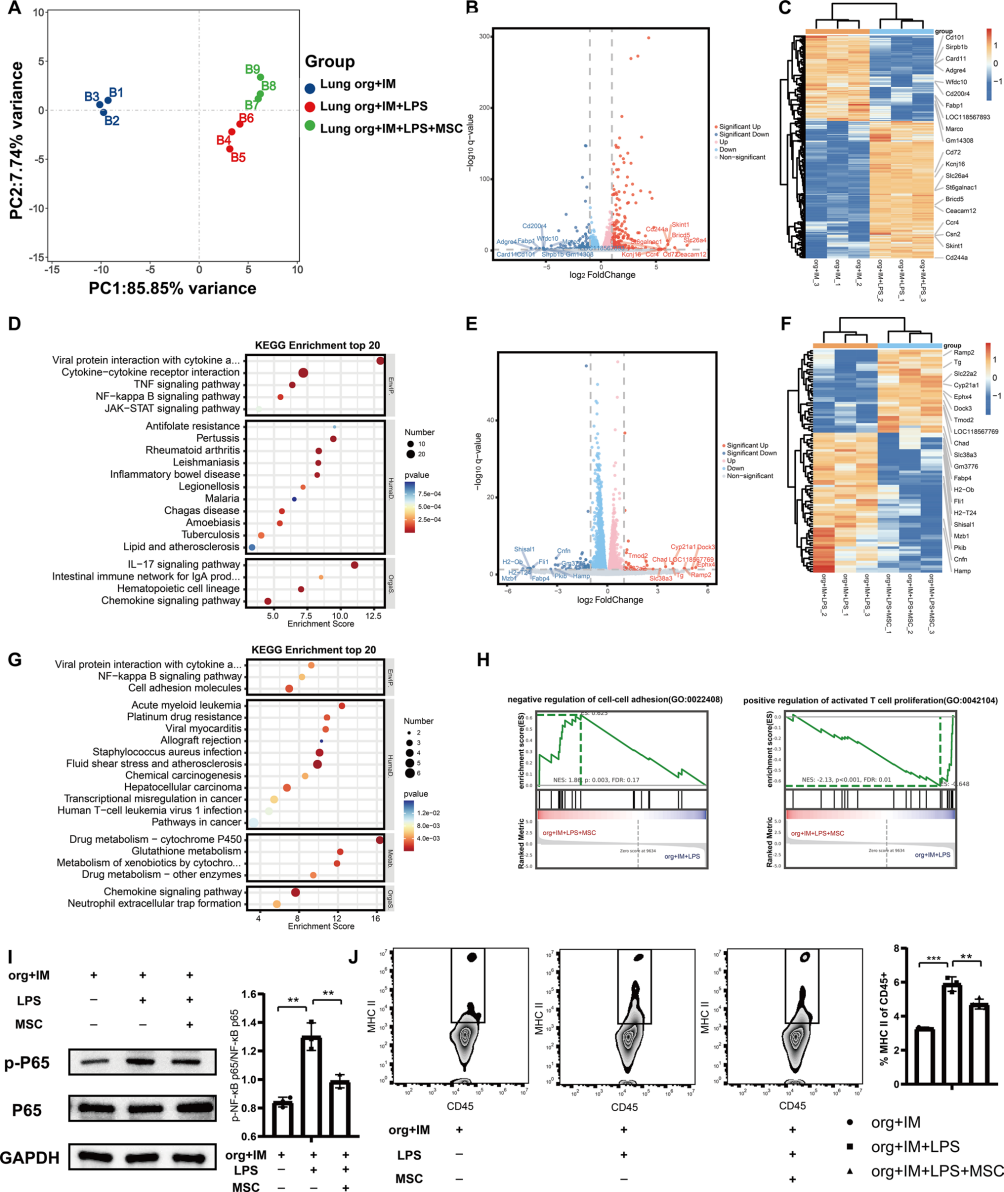
MSCs inhibit LPS stimulation-induced inflammatory responses and MHC II in IMs via the NF-κB pathway. **A** PCA plots of transcriptome expression in the lung organoid–IM group, LPS lung organoid–IM group, and LPS/MSC lung organoid–IM group. **B** Volcano plot for DEGs between lung organoid–IM group and LPS lung organoid–IM group. **C** Heatmap for DEGs between lung organoid–IM group and LPS lung organoid–IM group. **D** KEGG pathway enrichment analysis of the upregulated genes of lung organoid–IM group after LPS stimulation. **E** Volcano plot for DEGs between LPS lung organoid–IM group and LPS/MSC lung organoid–IM group. **F** Heatmap for DEGs between LPS lung organoid–IM group and LPS/MSC lung organoid–IM group. **G** KEGG pathway enrichment analysis of the downregulated genes of lung organoid–IM group after LPS stimulation by MSC treatment. **H** GSEA of DEGs of the downregulated genes of lung organoid–IM group after LPS stimulation by MSC treatment. **I** Representative images of western blotting of NF-kB P65 and p-NF-kB P65 in lung organoids-IMs subjected to LPS stimulation for 72 h while co-cultured with MSCs. Quantitative analysis of the relative expression level of p-NF-κB P65 and NF-κB P65 (*n* = 3). **J** Expression of MHC II in IMs after being treated with various formulations analyzed by flow cytometry (*n* = 3–4). Data are expressed as mean ± SD. **P < 0.01, ***P< 0.001

The results of PCA showed that the transcriptome expression patterns also differed among the three groups co-cultured with IMs (Fig. 7A). LPS stimulation upregulated lung organoids–IMs by 250 DEGs and downregulated them by 156 DEGs as shown in the volcano and heatmaps (Fig. 7B and C). Differential genes such as Sirpb1b, Wfdc10, ccr4, and cd244a that are associated with bacterial response, immune response, or inflammatory response were altered. KEGG enrichment analysis showed that upregulated genes were enriched in many pathways related to the immune system and inflammatory pathways, including TNF signaling pathway, NF-kappa B signaling pathway, IL-17 signaling pathway, and chemokine signaling pathway (Fig. 7D). Co-culture of MSCs resulted in the upregulation of 35 DEGs and downregulation of 59 DEGs in lung organoids–IMs (Fig. 7E and F). The downregulated DEGs were enriched in immune- and inflammation-related pathways such as NF-kappa B signaling pathway and chemokine signaling pathway (Fig. 7G). The WB results confirmed that MSCs suppressed LPS-induced NF-κB phosphorylation (Fig. 7H). GSEA showed that genes in the MSC co-culture group were significantly enriched in the negative regulation of cell–cell adhesion and not in the positive regulation of activated T cell proliferation (Fig. 7I). In addition, MSCs significantly downregulated the antigen-raising-related genes H2-Ob and H2-T24 in lung organoids–IMs (Fig. 7F), suggesting that they might achieve therapeutic efficacy by suppressing MHC-II expression. Flow analysis showed a significant increase in the proportion of MHC II in IMs after LPS stimulation, whereas MSCs inhibited its increase. These results suggest that for IMs, MSCs inhibit the LPS stimulation-induced inflammatory response in IMs in addition to their antigen-presenting ability through the NF-κB pathway.

## Discussion

Under normal physiological conditions, the lungs are the main organ of gas exchange in mammals and are in direct contact with the external environment, receiving a large number of external stimuli such as bacteria, viruses, dust, pollen, and particulate impurities. In addition, lung tissue contains a large number of immune cells, and is one of the important immune organs in the organism. Lung immune homeostasis is maintained by a network of tissue-resident cells that continuously monitor the external environment and, in a healthy state, are tolerant to harmless inhaled particles and mount an effective and rapid immune response to invading pathogens [35]. When the organism is in the midst of a severe infection, homeostasis in the lungs is difficult to maintain and immune dysregulation occurs, triggering pneumonia and resulting in severe ALI. ALI is a life-threatening inflammatory disease associated with cytokine storm, which leads to widespread excessive levels of proinflammatory cytokines in the lungs such as interleukins, TNF, and interferon; if not treated promptly and effectively, it can easily progress into acute respiratory distress syndrome (ARDS), leading to refractory hypoxemia and high mortality [25]. Currently, there is still a lack of effective treatment for patients with ALI/ARDS, and it is clinically important to find an effective treatment strategy.

The ability of organoids to mimic specific aspects of the 3D structure, cell type composition, and function of real organs, while maintaining the advantages of a simplified and easily accessible cell culture model, holds great promise for a range of biological and biomedical applications [19, 20]. In this study, we successfully isolated and cultured lung organoids from mouse lung tissues with a variety of lung tissue cell types, including basal cells, ciliated cells, secretory cells, club cells, and type II epithelial cells, which have the potential to better mimic lung organs. Compared with traditional 2D cell lines, the lung organoids in this study are richer in cell types, capable of multiple passaging, freezing, and resuscitation, and provide a better platform for simulating ALI disease models.

Furthermore, the traditional 3D culture system uses cells wrapped in matrigel to provide a 3D growth environment, but there are problems such as organoid wrapped in the gel, which is not fully exposed, hindered ability to receive external stimuli, and difficulty in achieving immune cell contact co-culture [36, 37]. In the present study, we improved the culture method by spreading the matrigel evenly on the bottom of the well plate, and the organoids were semi-exposed in the medium. The results showed that the added organoids were still able to grow into 3D structures after the matrigel was spread on the bottom. Interestingly, the cell arrangement was more complex, the basal cells originally wrapped around the periphery of the organoids appeared in the intraluminal portion, and the ciliated cells originally growing toward the lumen partly shifted to the extraluminal portion, which facilitated the organoids to receive the stimulation more directly.

Many of the organoids currently reported in the literature are still defective and do not mimic well the real situation in vivo, as they lack cellular components such as blood vessels, nerves, or immune cells [38, 39]. However, in this study, based on the improvement of lung organoid culture, we further added immune cells into the system to construct lung immune organoids. There are a large number of macrophages residing in the lung. The primary populations of pulmonary macrophages comprise AMs located in the airway lumen and IMs situated in the lung interstitium [40]. This study describes co-cultures of lung organoids and macrophages, and the inclusion of immune cells improves the accuracy of the organoids as an in vitro model, more closely replicating the physiological conditions of the organism. In addition, compared to organoids without immune cells, this model system is more conducive to studying pulmonary inflammatory diseases in the context of cellular diversity. Similarly, it has been reported in the literature that under LPS-induced inflammatory conditions, IL-1β, MCP-1, and TNF-α levels were significantly elevated in organoids containing induced hPSC-derived macrophages, whereas these levels were not detected in organoids alone [41]. Furthermore, our unshown data indicate that, apart from the presence of a small number of dead cell fragments in the lumen, the absence of macrophages resulted in no significant morphological changes resembling macrophage aggregation under LPS stimulation observed through light microscopy in the organoid. Therefore, we used this model to further delve into the in-depth mechanisms by which MSCs alleviate ALI through macrophages.

MSCs come from a variety of tissue sources and have been successfully isolated and cultured from tissues such as bone marrow, umbilical cord, placenta, or adipose tissue [42]. ClinicalTrials.gov data show that there are dozens of clinical treatment trial studies worldwide based on MSCs for the treatment of patients with different types of lung injury. MSCs are also potentially useful in the treatment of ALI due to their immunosuppressive effects [43]. Under exposed pathological conditions, MSCs migrate to the site of inflammation and modulate the immune response, producing various soluble factors and altering immune cell phenotypes and functions, which have great potential in immune-mediated disease treatment [44]. An increasing number of preclinical studies are exploring the efficacy of MSCs transplantation for ALI [45, 46].

The activation of macrophages in the lung plays a pivotal role in the inflammatory cascade [47]. The activated macrophages exhibit enhanced pinocytosis and phagocytosis capabilities, as well as the ability to synthesize and release ROS, cytokines, and other factors [48, 49]. ROS are an essential component of host defense against pathogens, and the body continuously produces and removes ROS to maintain a balance of their concentration in the body [50, 51]. Excessive formation or insufficient clearance of ROS leads to a wide range of pathological disorders, including ALI and ARDS [52–54]. Recent studies have demonstrated that the severity of novel coronavirus pneumonia (coronavirus disease 2019, COVID-19) is also associated with increased ROS formation [18, 55, 56]. AMs, IMs, neutrophils, endothelial cells, alveolar epithelial cells, and eosinophils are the main lung tissue ROS producers, which are associated with oxidative stress in ALI [57]. The results of the present study showed that LPS stimulated chemotactic migration of AMs and IMs to the peri-organoid area, generating large amounts of ROS and causing damage, but is inhibited by MSCs. Zhang et al. found that BMMSCs promote anti-inflammatory polarization of macrophages and produce low levels of ROS via exosomal delivery of miR-16 and miR-21 to attenuate SLE nephritis in lupus mice [58]. Similarly, it has been reported that hucMSCs significantly reverse senescence in HDFs by inhibiting ROS production and restoring the overexpression of oxidative and senescence markers through a paracrine effect [59]. Therefore, blocking excess ROS levels to alleviate oxidative stress is a promising strategy to reduce lung inflammation.

In addition, activation of inflammasome plays a key role in the formation of ALI [60, 61]. Previous studies reported that NLRP3 inflammasomes were significantly activated and mediated inflammatory damage in acutely injured lung tissues, and ROS could mediate the activation of NLRP3 inflammasomes [62–64]. IL-1β produced by NLRP3 inflammasome activation has strong immunomodulatory capacity and is a key proinflammatory factor that triggers an inflammatory response [65]. Transient expression of IL-1β in mouse lungs has been reported to cause severe symptoms of ALI [66]. In this study, the expression of NLRP3 inflammasome-associated components, NLRP3 and IL-1β, was significantly elevated, but treatment with MSCs significantly inhibited their expression, suggesting that MSCs may block the activation of NLRP3 inflammasomes by inhibiting the production of ROS, thus achieving therapeutic effects. Activation of NLRP3 inflammasome induces more neutrophil infiltration [67], and the macrophages are activated and transformed into M1-type proinflammatory macrophages [68], which secrete a variety of proinflammatory factors, such as IL-1β, TNF-α, and IL-6 [69], forming a vicious circle and amplifying inflammation. The results of this study showed that MSC co-culture significantly inhibited LPS stimulation-induced convergence phenomenon of AMs/IMs to organoids in the lung organoids–AMs/IMs co-culture system, inhibited their aggregation on the surface of the organoids and their entry into the lumen, inhibited the expression of inflammatory factor/chemokines, reduced the production of ROS, and inhibited the activation of NLRP3, thus reducing inflammatory responses and alleviating lung injury.

Once the lung microenvironment was stimulated, AMs were activated and immediately transformed into M1 phenotype, secreting large amounts of inflammatory mediators and promoting immune cell chemotaxis, thus exacerbating the inflammatory response of lungs, while M2 macrophages could inhibit inflammatory injury by releasing anti-inflammatory mediators [70, 71]. Xu et al.’s findings showed that the M1 phenotype predominated after LPS stimulation compared with the M2 phenotype [72]. Previous studies have shown that MSCs promote macrophage differentiation toward M2 and play an anti-inflammatory role in neonatal lung injury [73]. In our study, MSC transplantation alleviated AM recruitment in damaged lung organoids and modulated their M1/M2 phenotypic polarization, which is consistent with a previous study. Furthermore, MSC exosomes were effective in ameliorating experimental bronchopulmonary dysplasia and restoring lung function through macrophage immunomodulation [74]. NF-κB is involved in the immune-inflammatory response, and activated NF-κB is responsible for the conversion of M0 macrophages to M1 macrophages and cytokine production [75, 76]. Besides, NF-κB can act as a key regulator of cytokine expression and is closely associated with ROS production in macrophages and the induction of their apoptosis [77]. Our results show that MSCs can affect AM polarization via the NF-κB pathway, thereby inhibiting the inflammatory response and alleviating ALI.

Furthermore, macrophages can also participate in the immune-inflammatory response by increasing the expression of major MHC and CD molecules on the cell surface to process and present endogenous and exogenous antigens [78]. LPS induced an increase in MHC II expression in macrophage populations, suggesting that antigen-presenting cell activation can occur in the uterus of pregnant mice [79]. In the present study, we show that MSCs can reduce the proportion of MHCII in IMs to alleviate ALI. Similarly, previous studies have demonstrated that the MSCs reduce the expression of MHC II, CD40, and CD86 co-stimulatory molecules on mature DCs through an IL-6-dependent mechanism, which in turn leads to a decrease in T cell proliferation [80, 81].

Many beneficial effects of MSCs are mediated through their interaction with resident macrophages. AMs and IMs are both important components in regulating tissue homeostasis in the lung. Modulating their function can be used to prevent injury and promote repair in ALI. The interaction between MSCs and macrophages may occur through contact-dependent interaction or through paracrine-mediated secretion of signaling factors (nucleic acids, lipids, EVs, cytokines, chemokines, etc.) [82]. Currently, the regulatory effect of MSCs on macrophages mainly focuses on their influence on AMs, especially in regulating their M1/M2 polarization state, while there is less research on the mechanism of action on IMs. More research is clearly needed to determine the regulatory effects of MSCs on different macrophage subpopulations and to make these interactions predictable and modifiable to ensure favorable outcomes in clinical therapeutic applications.

In conclusion, macrophages in the lung can be classified into multiple subpopulations, and studying their contribution to the development of inflammatory lung injury has become an important area of research. In this study, we provide a novel culture strategy of lung organoid-macrophage model that are better able to receive stimuli to mimic disease, and thus explore the specific contribution of MSCs to the treatment of LPS-induced lung injury. The development of in vitro models with complex tissue structure and cell composition is conducive to further studying the mechanism and provides some guidance for the construction of a personalized in vitro model of patients. Compared with the LPS-stimulated group, MSCs altered M1/M2 polarization and downregulation of proinflammatory mediators, including inflammatory chemokines, inflammasomes, and ROS in lung organoids–AMs via the NF-κB pathway. In contrast, lung organoids–IMs exhibited significant downregulation of antigen presentation-related genes in addition to significant downregulation of the pro-mediators, suggesting that the effects of MSCs on these two macrophage subsets may play a different function in LPS-mediated inflammation.

### Supplementary Information

Below is the link to the electronic supplementary material.

Supplementary file1 (DOCX 1584 KB)

## Data Availability

The data for this study are available from the corresponding author upon reasonable request.
