# Supplementary material for: MSCs alleviate LPS-induced acute lung injury by inhibiting the proinflammatory function of macrophages in mouse lung organoid–macrophage model
Source: Cell Mol Life Sci. 2024 Mar 11;81(1):124. doi: 10.1007/s00018-024-05150-1 (PMC10927843; doi:10.1007/s00018-024-05150-1)

**Supplementary data**

**MSCs alleviate LPS-induced acute lung injury by inhibiting the proinflammatory function of macrophages in mouse lung organoid–macrophage model**

**Supplementary method**

**Isolation, culture, and identification of MSCs**

According to the literature, introduction by Zhu et al.^[1]^, and the improvements made by our research group, the isolation and culture methods of MSCs derived from mouse compact bones are as follows: The humerus, tibia, and femur of the limbs of C57BL/6 mice aged 2-3 weeks were carefully separated and the epiphysis at both ends of the bones were cut off and discarded with a blade, leaving only the backbone. The bone marrow hematopoietic stem cells in the bone marrow cavity were rinsed and the bone marrow fragments were digested by type II collagens and then placed in the mouse MSCs complete medium for static culture. It was observed under the microscope that the cell colonies formed by the cells crawling out of the bone fragments in the backbone of the culture plate were the primary MSCs derived from the isolated mouse compact bones. MSCs with 3rd to 5th-generation cell algebra are in the best state and will be studied in subsequent experiments.

**Identification of MSCs**

MSCs derived from mouse compact bone were cultured by osteogenic induction and differentiation, then stained with alizarin red dye, and observed the effect of osteogenic induction. After the induction differentiation culture, oil red O solution was used to stain and observe the impact of induction differentiation into lipids. After the induced differentiation culture of chondroblast, the effect of induced differentiation into cartilage was observed by staining with alisin blue solution. The surface markers of MSCs derived from mouse compact bone were identified by flow staining.

**RNA isolation and real-time quantitative reverse transcription polymerase chain reaction (qRT-PCR)**

Cells were collected as described previously and total RNA was extracted from the cells using the Trizol method. Reverse transcription was performed using HiScript III RT SuperMix Kit (Vazyme, Nanjing, China). Real-time qRT-PCR was performed in a volume of 20 μl using the ABI 7500 Real-Time PCR System and ChamQ Universal SYBR qPCR Master Mix Kit (Vazyme, Nanjing, China). The primer sequences are shown in the following table.

| **primer** | **sequence （5'-3'）** |
| --- | --- |
| CCL3-F’ | CCCAGCCAGGTGTCATTTTCC |
| CCL3-R’ | GCATTCAGTTCCAGGTCAGTG |
| CCL4-F’ | TTCTGTGCTCCAGGGTTCTC |
| CCL4-R’ | GAGGAGGCCTCTCCTGAAGT |
| CCL5-F’ | GTGCCCACGTCAAGGAGTAT |
| CCL5-R’ | GGGAAGCGTATACAGGGTCA |
| CXCL1-F’ | GCACCCAAACCGAAGTCATA |
| CXCL1-R’ | TGGGGACACCTTTTAGCATC |
| CXCL2-F’ | CGCCCAGACAGAAGTCATAG |
| CXCL2-R’ | TCCTCCTTTCCAGGTCAGTTA |
| IL-1β-F’ | TCATCTCGGAGCCTGTAGTGC |
| IL-1β-R’ | GCTGCTTCCAAACCTTTGACC |
| IL-6-F’ | AGGACTCTGGCTTTGTCTTTC |
| IL-6-R’ | CAATGGCAATTCTGATTGTATG |
| TNF-α-F’ | GCTCTGTGAAGGGAATGGGTGT |
| TNF-α-R’ | CCAGGTCACTGTCCCAGCATCT |
| GAPDH-F’ | GGTGAAGGTCGGTGTGAACGGA |
| GAPDH-R’ | GCAGAAGGGGCGGAGATGATG |

**Western blotting**

Cell precipitates were fully lysed with RIPA lysate containing two enzyme inhibitors, and the protein concentration was determined according to the procedure of the BCA kit. After adding with 5×SDS-PAGE protein upsampling buffer and boiled, the protein was run by a Mini-PROTEAN® Tetra Vertical Electrophoresis Cell (Bio-Rad Laboratories, Inc.) and then transferred to polyvinylidene difluoride membranes (Merck KGaA, Darmstadt, Germany). After incubation in blocking buffer (Beyotime Biotech Co., Ltd., Shanghai, China) at room temperature for 1 h, the membranes were incubated with primary antibodies overnight at 4°C, then washed three times in Tris-buffered saline with 0.1% Tween (TBST; Sangon Biotech) and incubated with secondary antibodies. After washing in TBST, the membranes were incubated with the Pierce™ ECL Western Blotting Substrate (Thermo Fisher Scientific) and detected with the ChemiScope Western Blot Imaging System (Clinx Science Instruments Co., Ltd., Shanghai, China). The antibodies are shown in the following table. ImageJ software (NIH, Bethesda, MD, United States) was used to analyze the image.

| **antibody** | **company** |
| --- | --- |
| Anti-NLRP3 | Abcam |
| Anti-NF-kB P65 | Cell Signaling Technology |
| Anti-p-NF-kB P65 | Cell Signaling Technology |
| Anti-TRIM15 | Invitrogen |
| Anti-GAPDH | Proteintech |
| horseradish-peroxidase-conjugated goat anti-rabbit IgG | Abcam |
| horseradish-peroxidase-conjugated rabbit anti-mouse IgG | Abcam |

**Reference**

[1] Zhu H, Guo Z K, Jiang X X, et al. A protocol for isolation and culture of mesenchymal stem cells from mouse compact bone. Nat Protoc, 2010, 5(3): 550-60.

**Supplementary Result**

**Culture and identification of MSCs**

After 3 weeks of induction of osteogenic differentiation, MSCs were stained with alizarin red S, and deep red calcified nodules of different sizes were observed (Supplementary Fig. S3B). After 4 weeks of induced lipogenic differentiation, MSCs were stained with oil red O, and a large number of red lipid droplets appeared in the cells (Supplementary Fig. S3C). After the MSCs induced chondrogenic differentiation were embedded, sliced, and stained with alisin blue, it was observed that the cartilage tissue contained acidic mucopolysaccharides stained blue (Supplementary Fig. S3D).

Single cell suspension was obtained and stained by flow cytometry antibody to detect the expression of specific markers of MSCs derived from mouse compact bone. Flow cytometry results showed that CD29, CD44, CD105 and Sca-1 were highly expressed, and CD11b, CD31, CD34, CD45, CD86 and MHC-II were low expression in MSCs isolated and cultured in this study (Supplementary Fig. S4).

The above results indicate that the mouse compact bone-derived MSCs extracted and cultured in this study have the growth morphology of MSCs, the differentiation potential of bone, lipid, and cartilage, and the expression characteristics of MSC markers{Jerkic, 2023 #1313}{Jerkic, 2023 #1313}.

**Supplementary Fig. S1**

Good growth capacity of lung organoids from different generations of murine lung tissue sources after passaging (P1, generation 1; P2, generation 2; P10, generation 10) (scale bar: 100 μm).


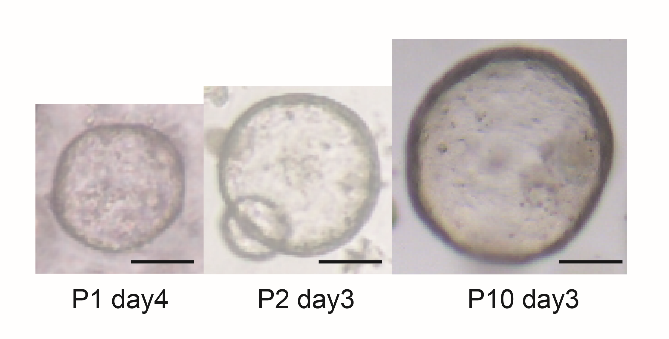


**Supplementary Fig. S2**

Immunohistochemical staining of lung organoids derived from mouse lung tissue verified the expression of α-tubulin, MUC5AC, SFTPC, CC10 and KRT5 cell type-specific markers (scale bar: 50 μm).


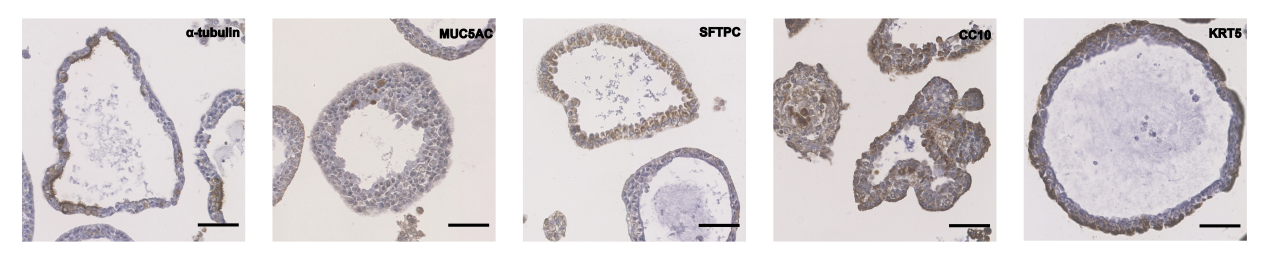


**Supplementary Fig. S3 Light microscopy, osteogenic, lipogenic and chondrogenic identification of MSCs.**

**(A)** Light microscopic observation of the growth morphology of MSCs showed a shuttle or fusiform shape (scale bar: 100 μm). **(B)** Identification of osteogenic differentiation of MSCs, dark red, variably sized calcified nodules were observed after alizarin red S staining (scale bar: 100 μm). **(C)** Identification of lipogenic differentiation of MSCs, a large number of reddish lipid droplets were observed after oil red O staining (scale bar: 100 μm). **(D)** Identification of chondrogenic differentiation of MSCs, blue acidic mucopolysaccharides were observed after alisin blue staining (scale bar: 100 μm).


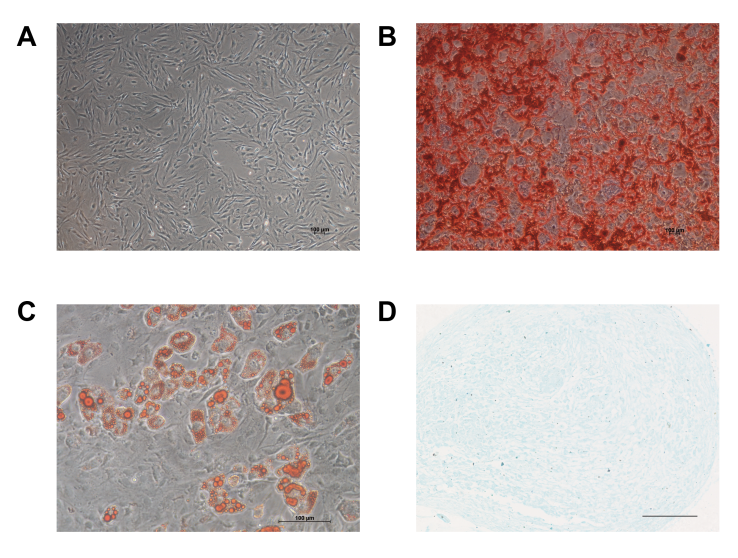


**Supplementary Fig. S4 Expression of surface-specific markers in MSCs.**

Flow cytometry analysis was performed to detect the expression of surface-specific markers in MSCs, which were highly expressed in mouse compact bone-derived MSCs: CD29 (98.8%), CD44 (96.2%), CD105 (63.9%), and Sca-1 (97.2%); and were lowly expressed in CD11b (4.26%), CD31 (4.89%), and CD34 (3.12%), CD45 (3.51%), CD86 (4.84%) and MHC-II (4.22%).


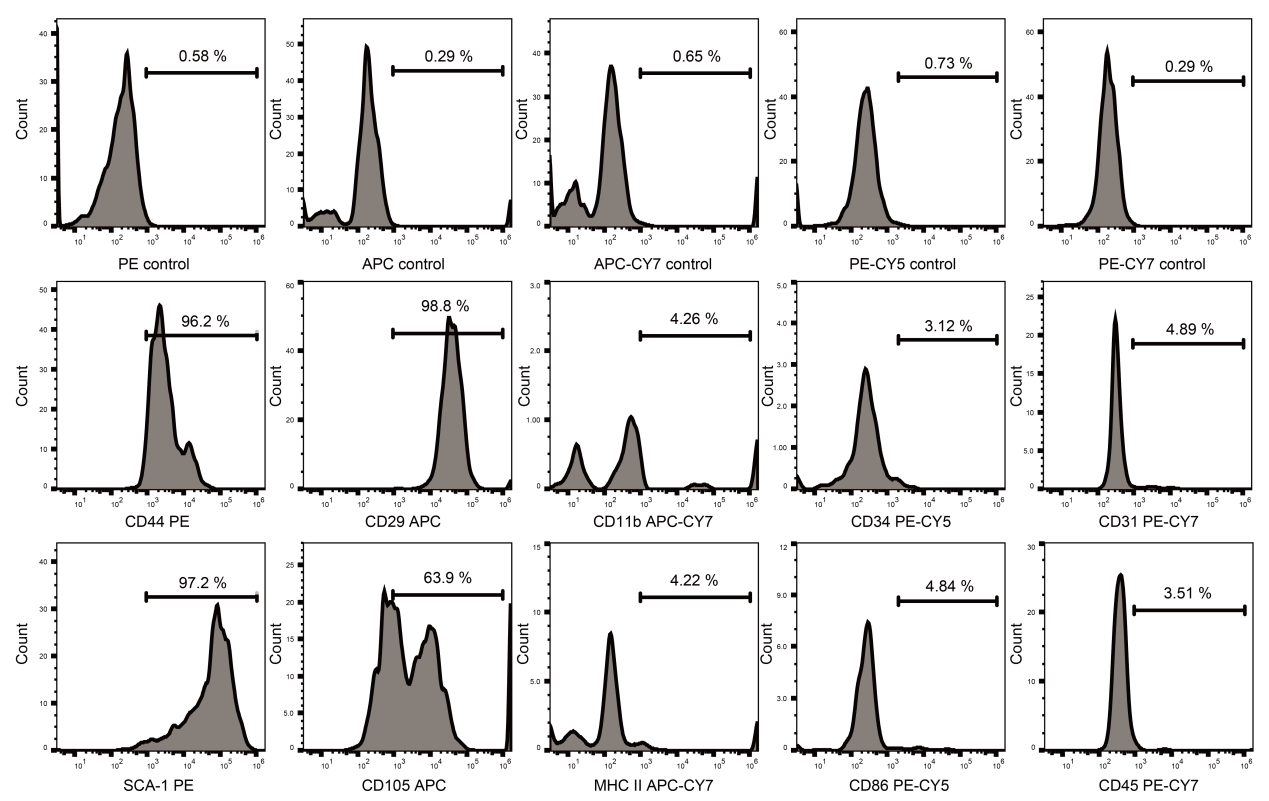

Supplement: Supplementary file 1 — Supplementary file1 (DOCX 1584 KB) [file 18_2024_5150_MOESM1_ESM.docx]
